# Concomitant Bacteremia in Adults With Severe Falciparum Malaria

**DOI:** 10.1093/cid/ciaa191

**Published:** 2020-02-28

**Authors:** Nguyen Hoan Phu, Nicholas P J Day, Phung Quoc Tuan, Nguyen Thi Hoang Mai, Tran Thi Hong Chau, Ly Van Chuong, Ha Vinh, Pham Phu Loc, Dinh Xuan Sinh, Nguyen Thi Tuyet Hoa, Deborah J Waller, John Wain, Atthanee Jeyapant, James A Watson, Jeremy J Farrar, Tran Tinh Hien, Christopher M Parry, Nicholas J White

**Affiliations:** 1 Hospital for Tropical Diseases, Ho Chi Minh City, Vietnam; 2 Oxford University Clinical Research Unit, Hospital for Tropical Diseases, Ho Chi Minh City, Vietnam; 3 Faculty of Tropical Medicine, Mahidol University, Bangkok, Thailand; 4 Centre for Tropical Medicine and Global Health, Nuffield Department of Medicine, Oxford University, Oxford, United Kingdom; 5 Quadram Institute Bioscience, Norwich, United Kingdom; 6 Clinical Sciences, Liverpool School of Tropical Medicine, Liverpool, United Kingdom; 7 Institute of Infection and Global Health, University of Liverpool, Liverpool, United Kingdom; 8 School of Tropical Medicine and Global Health, Nagasaki University, Nagasaki, Japan

**Keywords:** *Plasmodium falciparum*, malaria, severe malaria, bacteremia

## Abstract

**Background:**

Approximately 6% of children hospitalized with severe falciparum malaria in Africa are also bacteremic. It is therefore recommended that all children with severe malaria should receive broad-spectrum antibiotics in addition to parenteral artesunate. Empirical antibiotics are not recommended currently for adults with severe malaria.

**Methods:**

Blood cultures were performed on sequential prospectively studied adult patients with strictly defined severe falciparum malaria admitted to a single referral center in Vietnam between 1991 and 2003.

**Results:**

In 845 Vietnamese adults with severe falciparum malaria admission blood cultures were positive in 9 (1.07%: 95% confidence interval [CI], .37–1.76%); *Staphylococcus aureus* in 2*, Streptococcus pyogenes* in 1*, Salmonella* Typhi in 3, Non-typhoid *Salmonella* in 1*, Klebsiella pneumoniae* in 1, and *Haemophilus influenzae* type b in 1. Bacteremic patients presented usually with a combination of jaundice, acute renal failure, and high malaria parasitemia. Four bacteremic patients died compared with 108 (12.9%) of 836 nonbacteremic severe malaria patients (risk ratio, 3.44; 95% CI, 1.62–7.29). In patients with >20% parasitemia the prevalence of concomitant bacteremia was 5.2% (4/76; 95% CI, .2–10.3%) compared with 0.65% (5/769; 0.08–1.2%) in patients with <20% parasitemia, a risk ratio of 8.1 (2.2–29.5).

**Conclusions:**

In contrast to children, the prevalence of concomitant bacteremia in adults with severe malaria is low. Administration of empirical antibiotics, in addition to artesunate, is warranted in the small subgroup of patients with very high parasitemias, emphasizing the importance of quantitative blood smear microscopy assessment, but it is not indicated in most adults with severe falciparum malaria.

## Teaser

Blood cultures were positive in 9 of 845 sequentially studied Vietnamese adults with severe falciparum malaria. In contrast to children, concomitant bacteremia in adults with severe malaria is uncommon and does not warrant use of empirical antibiotics in all patients.

Malaria is associated with bacterial infection, but the relationship is complex [1]. In endemic areas, where the majority of symptomatic malaria occurs in children (ie, areas of moderate or high transmission), malaria is associated with an increased risk of bacteremia [2–9]. This risk is greatest in severe falciparum malaria. In a meta-analysis of 7208 children with severe malaria, included in 25 studies across 11 African countries, the mean prevalence of invasive bacterial infections was estimated to be 6.4% (95% confidence interval [CI], 5.81–6.98%) [8]. In these malaria-endemic areas it is now generally accepted that children presenting with severe malaria should receive broad-spectrum antibiotics in addition to parenteral artesunate [1, 10], as concomitant bacteremia cannot be excluded, and it is difficult to distinguish clinically between severe malaria and sepsis [11, 12]. A severely ill febrile child with a low parasitemia could have severe malaria or sepsis with incidental parasitemia. In contrast, in prospective studies of severe malaria conducted in low-transmission settings, where the majority of patients are adults, concomitant malaria and sepsis have been reported rarely—although there are few incidence data [13, 14]. Currently, empirical antibiotic treatment is not recommended in adults with severe malaria [1, 10]. However, 2 recent overlapping studies from Myanmar have challenged this recommendation. These studies found that 13 of 87 (15%) adult patients hospitalized with a diagnosis of malaria were bacteremic (ie, a substantially higher proportion than in African children with severe malaria) [15, 16]. Their report concluded that “clinicians should have a lower threshold for commencing empirical antibacterial therapy in adults diagnosed with falciparum malaria in these locations than is presently recommended.” Clearly, this is an important issue. We report a very large prospectively studied series of Vietnamese adults with strictly defined severe falciparum malaria in whom blood cultures were taken routinely in all patients on admission to the specialist treatment ward.

## METHODS

This investigation took place in the severe malaria ward of the Hospital for Tropical Diseases, Ho Chi Minh City, Vietnam, during 2 sequential studies of adult patients admitted with strictly defined severe falciparum malaria. The first, conducted between 1991 and 1996, was a double-blind comparison of intramuscular quinine and intramuscular artemether [17], and the second, conducted between 1996 and 2003, was a double-blind comparison of intramuscular artesunate and intramuscular artemether [18]. These 2 studies, reported previously in detail, were contiguous and all eligible patients were enrolled. Both studies were approved by the Ethical and Scientific Committee of the Hospital for Tropical Diseases, Ho Chi Minh City.

### Entry Criteria

Patients were included in the studies if they (or an accompanying relative) gave informed consent, they had asexual forms of *Plasmodium falciparum* on a peripheral-blood smear, were older than 14 years, were not in the first trimester of pregnancy, were not intravenous drug users, had received less than 3 g of quinine or 2 doses of artemisinin or a derivative in the previous 48 hours, were not allergic to the study drugs, and had 1 or more of the following: Glasgow Coma Scale score less than 11 (indicating cerebral malaria), anemia (hematocrit, <20%) with a parasite density greater than 100 000/μL, jaundice (serum total bilirubin, >2.5 mg/dL [50 µmol/L]) with a parasite density greater than 100 000/μL, acute kidney injury (urine output, <400 mL/24 hours, and serum creatinine, >3 mg/dL [250 µmol/L]), hypoglycemia (blood glucose, <40 mg/dL [2.2 mmol/L]), more than 10% parasitemia, and systolic blood pressure less than 80 mm Hg with cool extremities (indicating shock). These criteria are similar to the World Health Organization–endorsed definition [1], except that the anemia criterion is more stringent.

### Clinical Management and Procedures

On enrollment, patients were examined and weighed and baseline blood samples were taken for full blood count, clotting studies, biochemistry, arterial pH and blood gases, blood cultures, and thin- and thick-film malaria parasite counts. A full history was taken from the patient or attendant relatives and a full clinical examination performed including a detailed neurologic assessment. A urinary catheter was inserted. Patients were managed by a dedicated team according to standard recommendations [1]. Antimalarial treatment was started immediately with either artesunate, artemether, or quinine according to randomization as described previously [17, 18]. All patients were given isotonic saline initially, and fluid balance was then maintained with 0.9% saline or 5% dextrose in water. When necessary, a central venous catheter was inserted and the central venous pressure maintained at 5 cm of water. Blood was transfused if the hematocrit fell below 20%. Hypoglycemia was corrected with an injection of 50 mL of 30% dextrose and a subsequent maintenance infusion of 5% to 10% dextrose in water. Detailed clinical and nursing observations were recorded a minimum of every 4 hours for the first 24 hours. A diagnostic lumbar puncture was performed if the Glasgow Coma Scale score was below 14. Hemofiltration was started in patients with established renal failure. Patients with respiratory failure were ventilated. Acetaminophen was given for high fever (>39°C), and intravenous diazepam, intramuscular phenobarbital, and if necessary, intravenous phenytoin were given for convulsions. Antibiotics with no clinical antimalarial activity (ie, usually cefotaxime 2 mg/kg every 6 hours or ceftriaxone 2 g daily but not tetracyclines, macrolides, trimethoprim–sulfamethoxazole, or chloramphenicol) were given only if indicated clinically or cultures were positive, and confirmed enteric fever was treated with ofloxacin, but antibiotics were not started routinely.

### Microbiology

Between 5 and 15 mL of blood was taken for blood cultures (target, 10 mL). Between 1991 and 1997 a manual blood culture system was used [18]. Each 5-mL venous blood aliquot was inoculated into 50 mL of brain heart infusion broth (Tissue Culture Services, Perth, UK) with 0.05% sodium polyanethol sulfonate (Sigma, St Louis, MO). Blood culture bottles were vented and incubated at 35–37^o^C for 7 days. Blind subculture was performed at 24 and 48 hours and at 7 days or whenever physical growth was observed in the bottles. In September 1997, a BACTEC (Becton-Dickinson, Singapore) culture system was introduced. Aliquots of blood (5–8 mL) were inoculated into BACTEC plus aerobic bottles and then incubated for 5 days in a BACTEC 9050 automated analyzer. Bottles that gave a positive signal were subcultured.

Subcultures were plated onto fresh sheep blood agar and heated blood (chocolate) agar if *Haemophilus influenzae* or *Neisseria meningitidis* was suspected, and onto Sabouraud’s agar if a yeast or mold was suspected (all media supplied by Oxoid Unipath, Basingstoke, UK). Plates were incubated at 37^o^C in air (blood agar) or 5% CO_2_ (chocolate agar) for 48 hours or 30^o^C in air (Sabouraud’s agar) for 5 days. Organisms were identified by standard methods including API identification kits (Bio-Mérieux, Basingstoke, UK) when necessary. Specific antisera were used to identify *Salmonella* serogroups, including Vi for *Salmonella enterica* serovar Typhi (*S.* Typhi). *Staphylococcus epidermidis*, or other skin commensals were considered contaminants.

### Statistical Methods

Proportions were compared using Fisher’s exact test using Epiinfo (CDC, Atlanta, Georgia).

## RESULTS

Blood culture results were available for 845 adult patients admitted with severe falciparum malaria. Of these, 9 were positive for pathogens, a prevalence of 1.07% (95% CI, .37–1.76%). The organisms cultured were *S.* Typhi [3], non-typhoid *Salmonella* [1]*, Staphylococcus aureus* [2], Group A *Streptococcus* [1]*, H. influenzae* type b [1], and *Klebsiella pneumoniae* [1] (Table 1). An additional patient’s blood grew *Burkholderia cepacia*, but this was regarded as a contaminant, and the patient recovered uneventfully without receiving antibacterial treatment. The usual clinical presentation in these bacteremic patients was with the hepatorenal syndrome of fever, jaundice, and acute kidney injury accompanied by high parasitemia. One patient was unconscious (cerebral malaria).

**Table 1.** Clinical and Laboratory Features on Admission of Vietnamese Adults With Severe Falciparum Malaria and Concomitant Bacteremia

	Age and Sex	Weight, kg	Days of Fever	GCS	T, ^o^C	Antimalarial Drug	Antibiotic	Parasite Count/µL	Parasitemia, %	PCV, %	White Blood Count/µL (%N)	Plasma Creatinine, mg/dL	Total Bilirubin, mg/dL	Plasma Lactate, mmol/L	Organism Isolated	Outcome
1	47 M	45	3	14	37	Quinine	None	1 161 298	20.1	46	…	5.6	…	5.9	*Staphylococcus aureus*	Died
2	24 M	54	7	15	38.3	Artemether	Ofloxacin	224 322	4.7	38	7800 (74)	2.6	2.4	…	*Salmonella Typhi*	Survived
3	17 F	39	4	15	38.2	Artemether	Ofloxacin	501 144	11.4	35	4300 (67)	8.5	3.6	3.8	*S. Typhi*	Survived
4	24 M	65	15	4	38.5	Artemether	Ceftriaxone	39 564	0.9	35	9210 (60)	1	1.7	…	*S. Typhi*	Died
5	60 M	54	5	15	37.0	Artemether	Ofloxacin	128 740	4.1	25	3500 (75)	8.6	0.92	0.8	NTS	Survived
6	57 M	60	6	14	37.5	Artesunate	Ceftriaxone + oxacillin	38 685	0.7	44	8400 (79)	2.1	9.8	1.5	*S. aureus*	Survived
7	25 M	50	3	12	37.5	Artemether	None	906 958	24.9	29	14 850 (54)	5.6	…	7.0	*Haemophilus influenzae* B	Died
8	48 M	76	6	14	38.0	Artemether	Ceftriaxone + amikacin	1 235 904	24.6	40	6860 (53)	3.1	8.7	11.6	*Klebsiella pneumoniae*	Died
9	57 M	50	3	11	38.8	Artemether	Ceftriaxone	1 765 936	38.0	37	8640 (76)	3.0	8.5	6.0	*GAS*	Survived

Abbreviations: GCS, Glasgow Coma Scale; F, female; M, male; %N, % neutrophils; PCV, hematocrit; NTS, nontyphoid *Salmonella*; T, temperature; GAS, Group A *streptococcus*.

### Fatal Cases

Four of the 9 patients with severe malaria and concomitant bacteremia died, as discussed in the following:

1. A 47-year-old male farmer admitted with 20.1% parasitemia, jaundice, and shock died 2.5 hours after admission. Blood cultures subsequently grew *Staphylococcus aureus*.
2. A 24-year-old male farmer with a 7-day history of fever had generalized convulsions followed by coma on the day of admission. His parasitemia was 0.9%. He had clinical signs of pneumonia, for which he was given ceftriaxone, but he died 29 hours later without regaining consciousness. Blood cultures subsequently grew *S.* Typhi.
3. A 28-year-old male soldier with a 5-day history of fever was admitted with 24.9% parasitemia, jaundice, and acute renal failure. He died 16 hours later. Blood cultures subsequently grew *H. influenzae* type b.
4. A 48-year-old male builder with a 6-day history of fever presented with 24.6% parasitemia, hyperlactatemia, acute oliguric kidney injury, jaundice, pulmonary edema, and upper gastrointestinal bleeding. Hemofiltration was started immediately. His parasitemia rose to 54% within 8 hours of admission. He received artemether and, because sepsis was suspected clinically, he was given ceftriaxone. On day 2, blood cultures grew *K. pneumoniae,* and amikacin was added. After a protracted course he developed nosocomial pneumonia and he died 24 days later.

The overall mortality of patients with severe malaria but no concomitant bacteremia was significantly lower, 12.9% (108/836). The risk ratio for death in patients with concomitant bacteremia was 3.44 (95% CI, 1.62–7.29; *P* = .022).

### Risk Factors

The 9 patients admitted with severe malaria and concomitant bacteremia were slightly older (median age, 47 years; range, 17–60 years) than the other 836 patients (median age, 31 years; range, 15–79 years). They were also more likely to be hyperparasitemic; the median (range) parasite count was 501 144/μL (39 564 to 1 765 936/μL) compared with 81 766/μL (12 811 to 316 512/μL) in the patients with nonbacteremic severe malaria. Four of the 9 bacteremic patients had more than 20% parasitemia compared with 72 of 836 nonbacteremic patients (risk ratio, 5.16; 95% CI, 2.41–11.07; *P* = .0054). Thus, the prevalence of concomitant bacteremia in patients with more than 20% parasitemia was 5.2% (4/76; 95% CI, .2–10.3%) compared with 0.65% (5/769; .08–1.2%) in patients with less than 20% parasitemia, a risk ratio of 8.1 (95% CI, 2.2–29.5). Mortality in nonbacteremic patients with more than 20% parasitemia was 18% (13 of 72). Leukocytosis, which may also occur in very severe malaria infections, and other hematological or biochemical indices were not useful as indicators of concomitant bacteremia. Only 1 of the 8 bacteremic patients with a differential white blood cell count performed on admission had a neutrophilia.

### Community-acquired Bacteremias

Between 1991 and 2000, during which 90% of the patients in this series were recruited, *S.* Typhi was the predominant pathogen recovered from blood cultures taken in the hospital [18], comprising 41% (91/219) of isolates in 1991 and 25% (85/334) in 2000. Corresponding proportions for *S. aureus* were 14% (31/219) in 1991 and 10% (33/334) in 2000.

## Discussion

In this large prospective study of Vietnamese adults admitted to the hospital with strictly defined severe falciparum malaria the rate of concomitant bacteremia was low. This contrasts with large studies in African children with severe malaria in whom concomitant bacteremia is sufficiently common (more than 5 times more common than in adults in this series) [8], and the clinical distinction between severe malaria and sepsis is sufficiently difficult [11, 12] to warrant administration of antibiotics on admission to all children with a diagnosis of severe malaria [1, 10]. This low rate of concomitant bacteremia in adults supports current recommendations that empiric antibiotics should not be given on admission to adults with severe malaria unless there is clear evidence of a bacterial infection [1, 10]. The important exception is patients with very high parasite densities (>20% parasitemia) who were 5.2 (95% CI, 2.4–11.1) times more likely to be bacteremic. These high parasite counts in bacteremic patients, particularly the fatal cases, suggest that disease severity resulted primarily from malaria illness. The increased risk of bacteremia with very high parasitemias may reflect more-intense parasitized sequestration (eg, in the gut) and vital organ dysfunction [1] or, more specifically, host-phagocytic dysfunction resulting from the massive intravascular release of parasite cellular components and malaria pigment. *Salmonella* infections (particularly nontyphoid *Salmonella*) have been associated specifically with falciparum malaria infections in African children [2, 3, 5, 8, 19]. In this study, one-third of the bacteremias were with *S.* Typhi and it is noteworthy that *S*. Typhi was also the most common cause of community-acquired bacteremia identified in Ho Chi Minh City during this period [20, 21]. Although empirical antibiotics are not indicated on admission in adults with severe malaria unless they have very high parasite counts, antibiotic treatment may well be needed subsequently in patients who deteriorate [1], as nosocomial bacterial infections are relatively common following admission in severely ill patients.

These results contrast markedly with a recent study from Myanmar in which 15% (13 of 87) of adults hospitalized with a primary diagnosis of malaria were bacteremic [16]. Malaria transmission in both countries is generally low and seasonal. However, the Myanmar patient characteristics were very different from those of the Vietnamese adults with strictly defined severe falciparum malaria. Only some of the Myanmar patients may have had severe malaria, semi-quantitative malaria parasite counts were generally low (and were significantly lower in bacteremic than in nonbacteremic patients), and many had neutrophil leukocytosis, all suggesting a primarily bacterial illness. The prevalence of bacteremia in the Myanmar adult patients was 23 times higher than in the Vietnamese adults with strictly defined severe falciparum malaria and less than 20% parasitemia (15% vs 0.65%). Even in the 3 Myanmar fatal cases parasite counts were low (recorded as 1+ in 2 patients, and 2+ in 1 patient), whereas all the parasite counts of the Vietnam bacteremic patients would have scored 4+ in the semi-quantitative system—and with quantitative counts, 4 had more than 20% parasitemia (of whom 3 died). The low overall mortality in the Myanmar series of 3.4% (3/87) was attributed to early use of antibiotics; yet, most of the Vietnamese patients did not receive antibiotics. It is therefore very unlikely that a high proportion of them had covert bacterial septicemia. The simplest and most probable explanation for the marked difference between the 2 studies relates to the primary diagnosis. The Vietnam patients undoubtedly had severe falciparum malaria as their primary condition, with bacteremia occurring late in the course of their illness, whereas it is likely that the bacteremic Myanmar patients had bacterial sepsis as their primary condition, and their malaria parasitemia was incidental (ie, their fever and illness were caused by their bacterial infections and not malaria). This would explain the apparent high prevalence of bacteremia, their very low parasite counts, and their neutrophil leukocytosis. Asymptomatic parasitemias are common in malaria-endemic areas. It is understandable that an ill febrile patient with a positive malaria smear would be considered to have malaria if there was no obvious focus of bacterial infection. However, severe falciparum malaria results from a current or previous large sequestered parasite burden [1], whereas incidental parasitemia is associated with parasite burdens that are many orders of magnitude lower [22]. These 2 very different syndromes, requiring different management, can be distinguished by quantitative malaria parasite counts and by other parasite burden indicators such as the proportions of neutrophils containing malaria pigment, the stage of malaria parasite development, and plasma concentrations of *Pf*HRP2 or *P. falciparum* DNA [23–26]. As parasite counting, staging, and neutrophil pigment assessment can all be done rapidly on admission thin- and thick-film blood films, this emphasizes the value of experienced microscopy in the assessment of patients hospitalized with malaria. The interval from taking the blood smear to completing the thin-film parasite count can be as little as 5 minutes [27]. Semi-quantitative counts using the old “plus” or “cross” system are unreliable and are no longer recommended by the World Health Organization [28]. They are particularly unsuited for patients hospitalized with malaria, as the maximum semi-quantitative count of 4+ (>10 parasites in 1 thick blood film oil-immersion high-power field) encompasses parasitemias ranging from less than 1% to 100%. Misdiagnosis also contributes significantly to the high rates of concomitant bacteremia and “severe” malaria reported in African children [25] and explains many of the apparent associations between both falciparum and vivax malaria and a variety of unrelated conditions. The high prevalence of asymptomatic parasitemia in endemic areas means that many patients admitted to the hospital with other conditions will be labeled as having malaria. In a retrospective review of 400 adult patients with severe imported malaria admitted to 45 French intensive care units, 9 (2.3%) were bacteremic and 1 had candidemia on admission [29]. However, the patients in France were substantially older (median age, 45 years) than the Vietnamese adults in this series, 7.3% of patients had immune deficiencies, and 14.3% had 1 or more comorbidities.

Although this study in Vietnamese adults was long, large, and detailed, it has several limitations. Empirical use of antibiotics before admission to the hospital is common in Asia (and was reported in 35% of the Myanmar series) and could have obscured some bacterial infections. However, prior antibiotic use was unusual in the low-income rural population at risk of malaria in Vietnam between 1991 and 2003, so is unlikely to be a significant confounder. Blood culture is intrinsically insensitive, so bacteremia was probably underestimated. The target blood volume cultured (10 mL), which was the same in both the Myanmar and Vietnam series, is not maximally sensitive [30, 31]. It is also noteworthy that 6 of the 9 positive blood cultures were obtained following the change to an automated blood culture system, so the earlier manual culture system may have been less sensitive. However, the Vietnamese patients with severe malaria were not given routine antibiotics after admission, yet 87% survived, and most deaths were clearly attributable to severe falciparum malaria. It seems very unlikely that these various differences could account for the marked discrepancy between this and the Myanmar series, particularly as many of the Myanmar patients did not have strictly defined severe malaria.

High parasitemia was clearly a risk factor for concomitant bacteremia. Of the 76 patients with more than 20% parasitemia (9% of the total), 4 (5.2%) were also bacteremic. This group has a high mortality, so giving broad-spectrum antibiotics empirically to patients with very high parasitemias is justified. Thin blood smear assessment should be performed on all patients admitted with severe falciparum malaria. Overall, however, in contrast to children in areas of higher malaria transmission, the incidence of concomitant bacteremia in adults with severe malaria is low and does not warrant the use of empirical antibiotics in all patients.

## Notes

***Author contributions*.** N. J. W. designed the study; N. H. P., N. P. J. D., P. Q. T., N. T. H. M., T. T. H. C., L. V. C., H. V., P. P. L., D. X. S., D. J. W., J. J. F., and T. T. H. conducted the clinical studies; N. T. T. H. and C. M. P. conducted the laboratory studies; A. J., J. W., and N. J. W. analyzed the data. All authors contributed to the study implementation, interpretation, and reporting

***Acknowledgments*.** The authors are grateful to the nurses and doctors of the malaria ward and the staff of the microbiology laboratory of the Hospital for Tropical Diseases, Ho Chi Minh City. Deidentified individual patient data from these 2 sequential studies are available on request to the Oxford University Clinical Research Unit data access committee.

***Financial support*.** This work was supported by the Wellcome Trust through serial grants to support the Clinical Research Program.

***Potential conflicts of interest.*** The authors: No reported conflicts of interest. All authors have submitted the ICMJE Form for Disclosure of Potential Conflicts of Interest.
